# Fast Healthcare Interoperability Resources (FHIR)–Based Interoperability Design in Indonesia: Content Analysis of Developer Hub’s Social Networking Service

**DOI:** 10.2196/51270

**Published:** 2025-04-21

**Authors:** Lukman Heryawan, Yukiko Mori, Goshiro Yamamoto, Naoto Kume, Lutfan Lazuardi, Anis Fuad, Tomohiro Kuroda

**Affiliations:** 1 Department of Computer Science and Electronics, Faculty of Mathematics and Natural Sciences, Universitas Gadjah Mada, Sekip utara BLS 21, Sleman, 55281, Indonesia, 62 81228944870; 2 Division of Medical Information Technology and Administration Planning, Kyoto University Hospital, Kyoto University, Kyoto, Japan; 3 Preemptive Medicine and Lifestyle Related Disease Research Center, Kyoto University Hospital, Kyoto, Japan; 4 Health Policy and Management, Faculty of Medicine Public Health and Nursing, Universitas Gadjah Mada, Sleman, Indonesia

**Keywords:** interoperability, Fast Healthcare Interoperability Resources (FHIR), COVID-19, Satusehat, Indonesia, interoperability design, social networking, health information, content analysis, private clinic

## Abstract

**Background:**

Interoperability in health care is a critical aspect for the exchange of health information. The Fast Healthcare Interoperability Resources (FHIR) framework has become widely adopted to provide interoperable data exchange in the health care industry. The COVID-19 pandemic has demonstrated the significance of interoperable data in tracking patients who have contracted the virus and keeping track of the vaccinated population. Indonesia is one of the many countries that have implemented interoperable data systems to track patients with COVID-19, and it has aspirations to expand the system to other use cases, particularly in the primary health care setting. The primary health care providers in Indonesia include *Puskesmas* (community health centers) and private clinics.

**Objective:**

To promote interoperable health data exchange in the primary health care sector, the Indonesian government has launched the Satusehat project. The goal of the Satusehat platform is to make health data in Indonesia interoperable and exchangeable between health care organizations, particularly *Puskesmas* and private clinics.

**Methods:**

For a successful implementation of the Satusehat platform in *Puskesmas* and private clinics, it is crucial to understand the challenges that may arise. This study analyzed the pain points of the Satusehat platform based on a content analysis of the Satusehat Social Networking Service Telegram group messages. The study revealed the pain points and suggested existing approaches to address them, which can be used as a proposed design of interoperability for *Puskesmas* and private clinics, making it easier for these organizations to adopt the Satusehat platform.

**Results:**

The pain points identified in this study include issues with the FHIR server, problems with FHIR profile selection, and the mapping of electronic medical record data into standardized data, such as mapping into the Systematized Nomenclature of Medicine Clinical Terminology. The results show that the value of the mapping issue is 37, profile issue 9, and server issue 61. Among the 3 categories, server issues had the highest population, followed by mapping issues and then profile issues. To address these issues, the study proposed practical approaches, including a federated architecture for the FHIR server instead of a centralized architecture, an FHIR writer and FHIR viewer system inspired by the Standardized Structured Medical Record Information eXchange system in Japan, and an FHIR conversion framework that integrates with our FHIR writer and FHIR viewer system.

**Conclusions:**

These proposed solutions can help resolve the pain points identified in the study and help the advancement of the Satusehat platform implementation in *Puskesmas* and private clinics in Indonesia. We believed that the proposed solutions have potential to be adopted to other countries with similar issues when conducting nationwide project in health care interoperability design.

## Introduction

### Background

Health information exchange (HIE) involves the transfer of electronic health data between different health information systems, including the electronic medical record (EMR) system, the electronic health record (EHR) system, and the personal health record (PHR) system. Notably, when the data are controlled by a health facility, an EMR system transitions into an EHR system. Conversely, if the data are managed by the patient or consumer themselves, it transforms into a PHR system [1]. To facilitate this exchange, HIE requires a data exchange or data interoperability framework that provides a standardized format for transferring health data [2]. One of the organizations that provides such interoperability frameworks is Health Level Seven (HL7) [3]. HL7 is a nonprofit organization that develops interoperability frameworks, such as HL7 FHIR (Fast Healthcare Interoperability Resources). It is a newer standard that provides a modern and flexible approach to exchanging health information. It uses a Representational State Transfer web services approach and supports both structured and unstructured data [4]. There have been several studies and reports on the implementation of EHR systems using the FHIR standard in different countries. One study, focused on the implementation of an FHIR-based EHR in Japan, highlighted some of the challenges faced during the implementation process, including lack of standardization and compatibility between different systems, difficulty of data migration, and the need for improved communication and cooperation between health care providers [5]. Another study provided the significance of standardization, data quality, and the need for technical support. The study also offered recommendations for improving the implementation and adoption of FHIR-based EHR systems [6,7].

**Figure 1.**
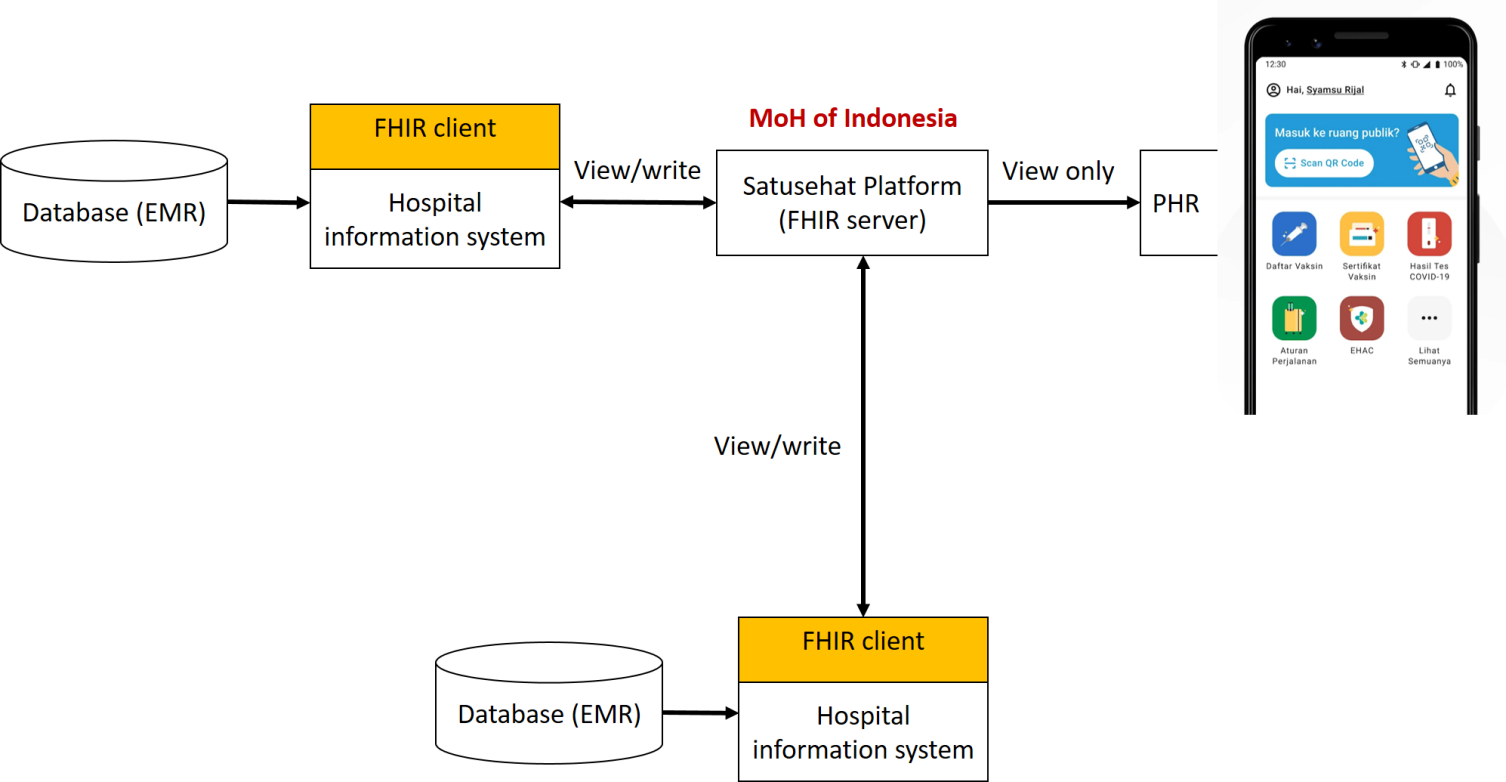
Satusehat platform’s architecture. PeduliLindungi app as a PHR is integrated to FHIR server with view only. On the other hand, hospital information system with data from EMR could view or write with other health care applications. The FHIR server which is handled by the Ministry of Health acts as a bridge between health care digital instances. EMR: electronic health record; FHIR: Fast Healthcare Interoperability Resources; MoH: Ministry of Health; PHR: personal health record.

The Ministry of Health (MoH) manages the EHR system in Indonesia, while the EMR system is independently managed by medical providers, such as hospitals, *Puskesmas* (community health centers), and private clinics. The PHR system, on the other hand, is provided by the MoH to the citizens of Indonesia [8]. In 2022, the expansion of EMR implementation to encompass all types of health care facilities was enacted through MoH decree number 24/2022. This regulation is a part of the digital health transformation blueprint released by the MoH [9]. A significant driver in the adoption of EMR, EHR, and PHR in Indonesia has been the COVID-19 pandemic. The PeduliLindungi app, developed as a tool for tracking, tracing, and monitoring during the pandemic, has become a crucial part of this initiative. This app has been used by more than 100 million users, making it interoperable with hundreds of laboratories and 17 telemedicine service providers. Most recently, through the MoH of the Republic of Indonesia decree number HK.01.07/MENKES/1559/2022, health data management is now under the MoH. Data from the PeduliLindungi app have been migrated to the Satusehat platform, as shown in Figure 1, which will integrate the PHR system and EMR system into a single nationwide EHR system [10]. The Satusehat Mobile platform enables the public to track COVID-19 vaccinations, immunization records, and medication information from EMRs of various health care facilities connected to this platform, using the FHIR HL7 standard. Furthermore, the EMR systems will be able to exchange and share data with ease through the Satusehat platform.

Legal basis for the implementation of the Satusehat system in Indonesia has been further strengthened with the enactment of the Health Law number 17/2023. This legislation specifically references the National Health Information System, which is interpreted as a system based on the Satusehat platform. The forthcoming derivative laws are expected to lay out the structural and operational details, ensuring a coherent and efficient implementation of these systems across the country.

**Figure 2.**
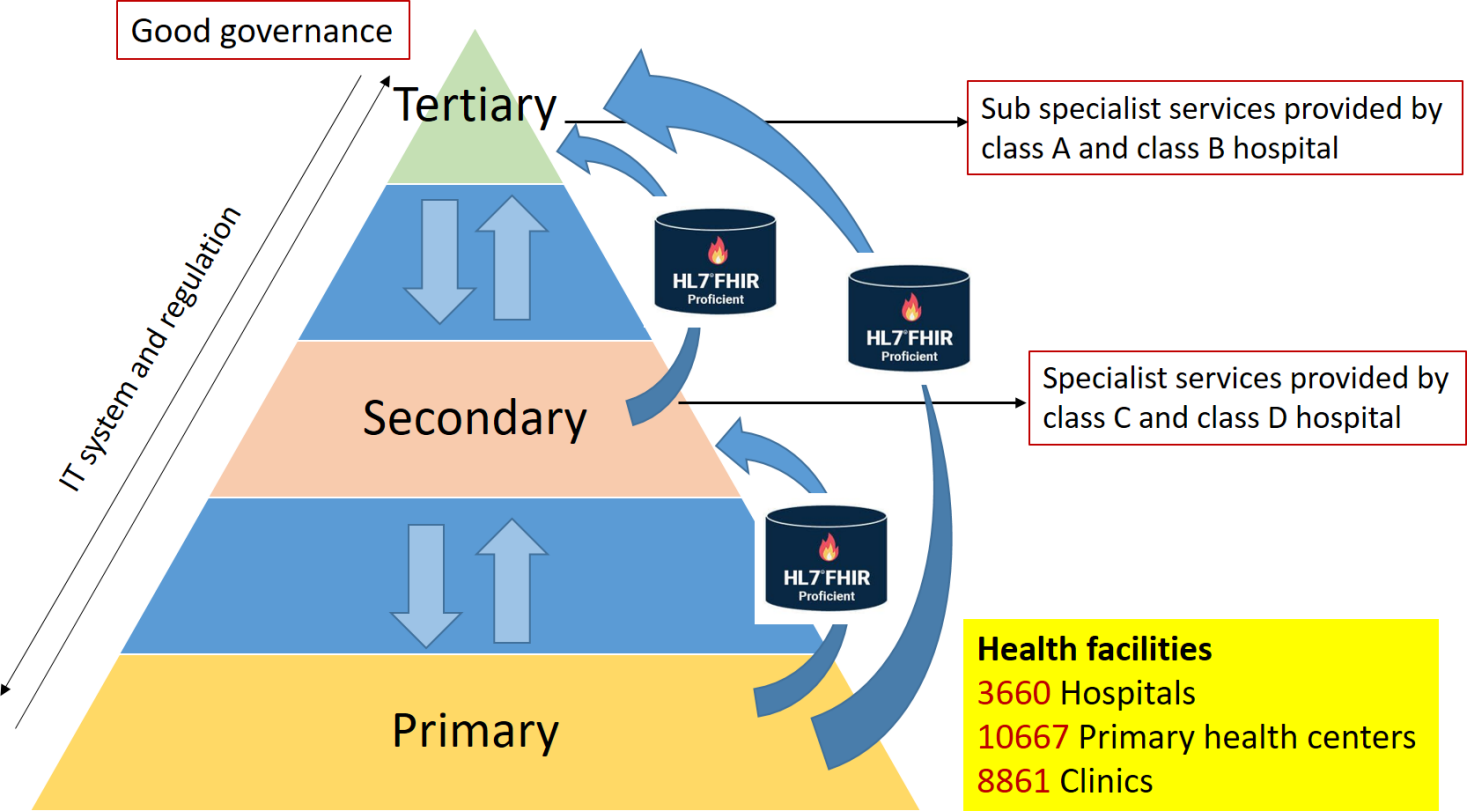
Indonesia’s health care tiered referral system. Primary care facilities such as *Puskesmas* or private clinics as the base and entry point of health care service. Patient progress or referred to higher level, including secondary of tertiary facilities, if needed. This system is implemented with HL7 FHIR between tiers, thorough IT system, and regulation. HL7 FHIR: Health Level Seven Fast Healthcare Interoperability Resources.

The Satusehat platform represents the cornerstone of Indonesia’s nationwide EHR system [11]. This platform aims to facilitate the exchange and sharing of health data, ultimately leading to better patient care and outcomes. In Indonesia, the Satusehat platform places a strong emphasis on primary health care. This is caused by the referral health care system in place, where most patients’ health data begin with their first visit to a primary health care facility such as a *Puskesmas* or private clinic. These facilities are typically the first point of contact for patients seeking medical attention. If the primary health care facilities are unable to provide the necessary treatment or care, the patient will be referred to a higher level of care, such as a hospital. This referral system is depicted in Figure 2, which shows the progression of patients through different levels of care within the Indonesian health care system [12].

**Figure 3.**
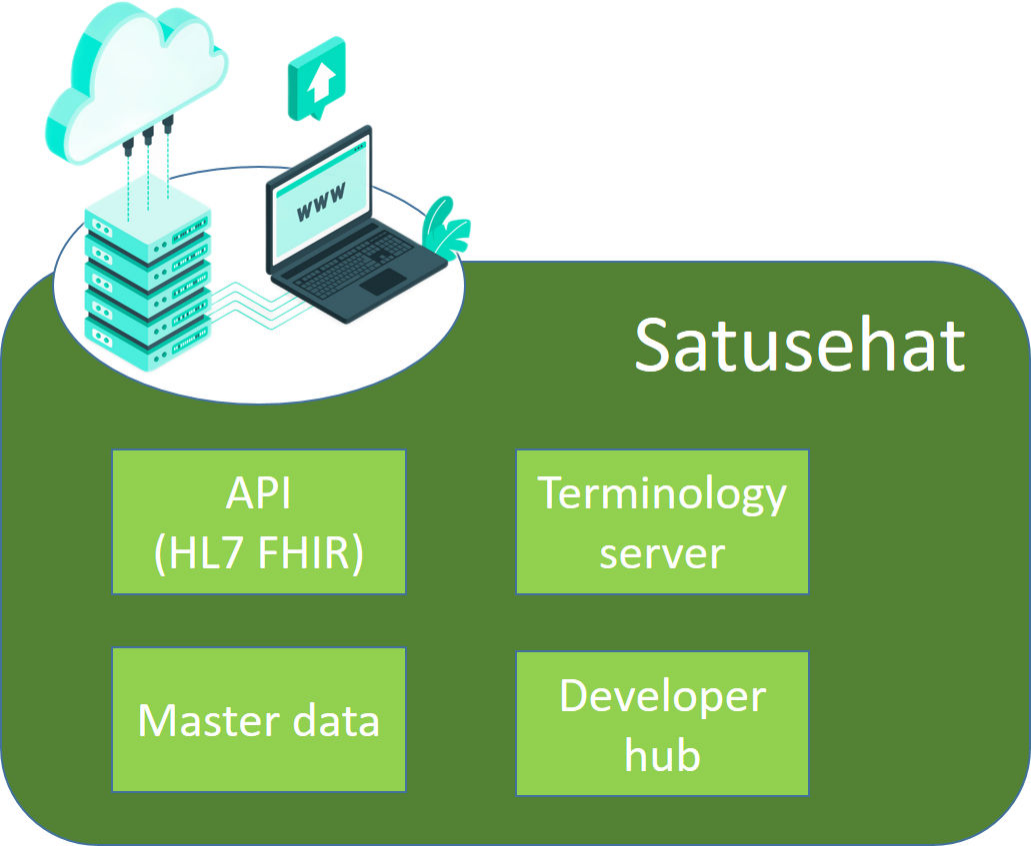
Satusehat platform components that consist of FHIR server, terminology server, master data server, and developer hub. FHIR is responsible for health data exchange, terminology server provides diagnoses terminology standardization, master data stores patient and health care instances data (practitioners and infrastructures), and developer hub is for support center for developers when using FHIR application programming interface. HL7 FHIR: Health Level Seven Fast Healthcare Interoperability Resources.

The Satusehat platform consists of 4 primary components: the FHIR server, the terminology server, the master data server, and the developer hub as shown in Figure 3. The FHIR server is responsible for providing an HL7 FHIR application programming interface (API) for health data exchange. The terminology server serves as a database, providing standardized data references for diagnoses using ICD-10 (*International Statistical Classification of Diseases, Tenth Revision*), observations using Logical Observation Identifiers Names and Codes (LOINC), and other clinical terms using Systematized Nomenclature of Medicine Clinical Terminology (SNOMED-CT). The master data server is a database that contains the nationwide identification numbers of patients or citizen ID, practitioner data, health facilities data, pharmacies data, financing data, and health service data. The developer hub provides a sandbox environment for developers to test the FHIR API offered by the MoH. Developers can experiment and test data exchange scenarios, such as immunization, using the provided FHIR API. The developer hub also includes community tools, such as a video-sharing platform, a website, and a Telegram group, to provide support and assistance to developers while they test the Satusehat FHIR API [13].

To ensure a successful implementation of the Satusehat platform in *Puskesmas* and private clinics, it is crucial to identify potential pain points in integrating EMR into the platform from a developer’s perspective. Understanding the underlying architecture and structure of the FHIR standard is necessary for effective integration with EHR systems [14]. Another reason for pain points is the lack of resources, as implementing FHIR standards can be resource-intensive, requiring time and expertise. Developers may face constraints in terms of budget and man power, which can make it difficult to effectively implement the standard [15]. In addition, the lack of support from other stakeholders, including health care providers and technical experts, can also pose challenges for developers in implementing FHIR standards [16]. The Indonesian MoH is currently in the trial phase of the Satusehat platform. The Digital Transformation Office (DTO) of the MoH conducts workshops and training sessions for EMR system developers and vendors [17]. DTO also provides a website that provides step-by-step tutorials on how to connect and exchange data from EMR systems to the Satusehat platform using the FHIR framework, including the standardized terminologies used for mapping health data to standardized terminologies, such as LOINC for observations, *ICD-10* for diagnoses, and SNOMED-CT for other health terminologies. To provide interactive support, DTO also provides a Telegram group that developers can join to ask for support when integrating their EMR systems into the Satusehat platform. Based on the activities in the Telegram group, which mostly involve questions about how to integrate EMR systems into the Satusehat platform, we conclude that there are some pain points when integrating EMR systems into the Satusehat platform. This conclusion came from analysis through the Telegram group including a question and answer session with the DTO representative.

### Objectives

The purpose of this study is to identify the challenges and difficulties encountered by developers that are present in situ during the integration process of EMR systems into the Satusehat platform. To accomplish this, we will analyze the communication content in the Telegram group of the Satusehat developer hub, which serves as an interactive community platform for developers. By exploring the pain points communicated by the developers in this forum, we aim to gain a deeper understanding of the barriers and obstacles encountered during the integration process. Once the pain points have been revealed, the study proposes practical approaches that can be used to ensure a successful integration into the Satusehat platform. These approaches are then combined to form an interoperability design of an FHIR-based system for EMR in *Puskesmas* and private clinics. The study is organized into 4 sections. The first section introduces the Satusehat platform, related work, and how pain points will be revealed. The second section describes the content analysis methods used in the study. The third section presents the results, and the fourth section discusses the practical approaches that will be proposed as an interoperability design to ensure a smoother integration into the Satusehat platform.

## Methods

### Study Design

**Figure 4.**
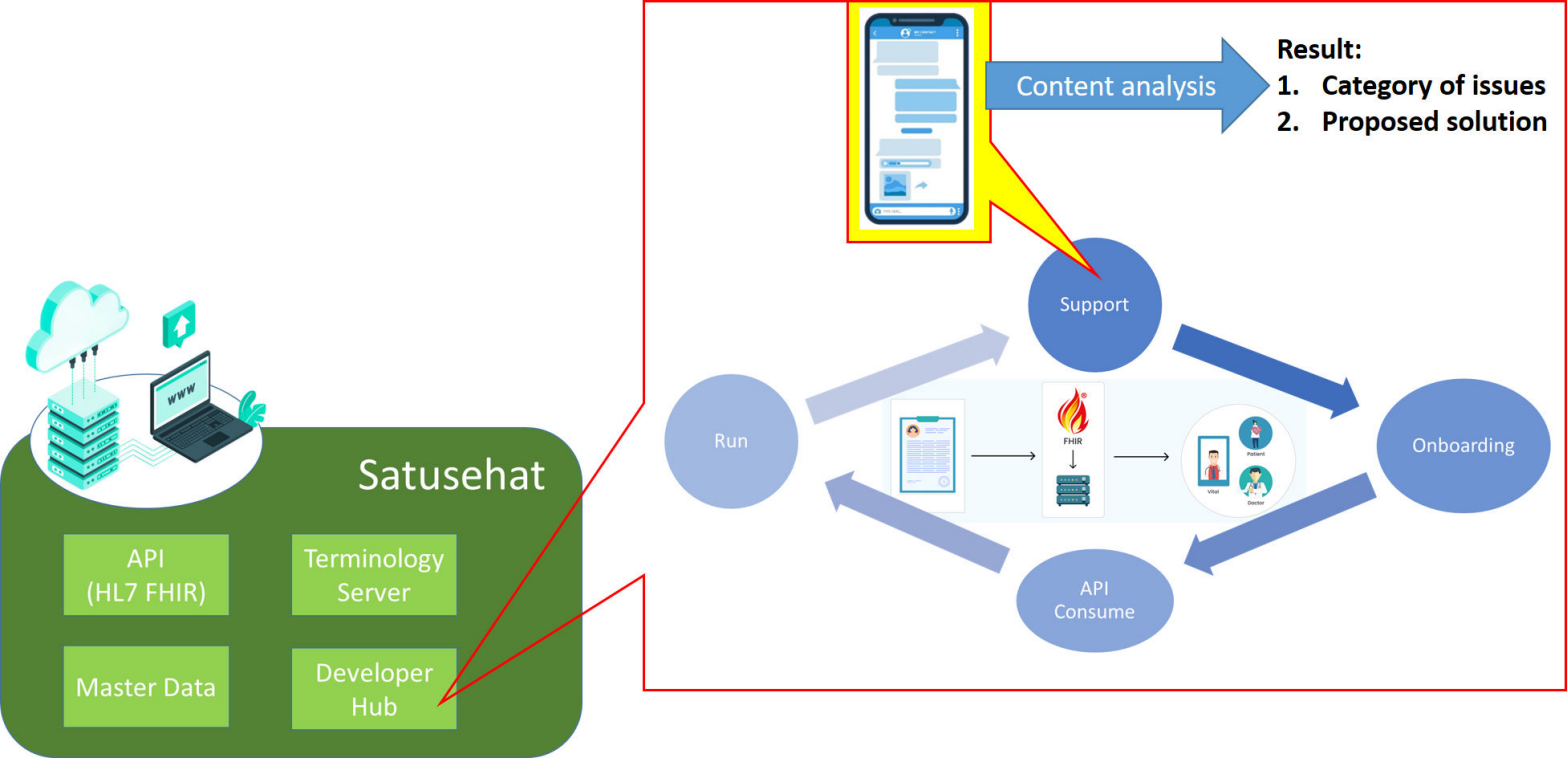
The approach method of the study. Given the developer hub, as the support and assistance forum for developers to test FHIR, the Telegram group chat content is extracted. Then, content analysis is conducted that resulted in categorization of issues and proposed solutions for each issues. API: application programming interface; HL7 FHIR: Health Level Seven Fast Healthcare Interoperability Resources.

To uncover the pain points that are addressed in the developer hub, we conducted a content analysis of the Telegram group’s contents. We decided to conduct the analysis to the specific telegram group because the group is officially managed by the DTO. It is the most active group that contains developer discussions about EMR integration to the Satusehat platform. Content analysis is a research method used to make inferences by interpreting and coding written, spoken, or visual material [18]. The analysis was conducted at a Telegram group that consists of 1067 active members to this day. This study focused on analyzing conversations from December 20, 2022, the date the group was created, until January 29, 2023. The end date was chosen because the amount of extracted data is sufficient to support the experiment, which is content analysis. Extending the time span of chat extraction could potentially enhance the volume of data collected and strengthen the robustness of our conclusions, as the amount of data sampled in an experiment highly depends on the subject of the study [19]. In this instance, our focus was on Telegram as a social network service. A total of 219 chats were chosen during this period. However, we selected only 107 chats related to our research purpose. These chats primarily involved developers discussing issues encountered while integrating their EMR systems with the Satusehat platform using FHIR standards. In our research, we focused on issue-related chats, such as “http server error 500,” “what FHIR profile should be used for chief complaints,” and “SNOMED-CT or LOINC for mapping blood glucose.” We excluded nonissue chats that contained simple responses such as “Ok” or “thank you.” To collect the chats, we used the Telegram menu to export them into HTML files, which we later transferred to an Excel file. The collected chats were later manually labeled by 2 of the researchers (LH and ADW), who are trained to categorize the data according to agreed and specific rules for these issue-related chats. The labeler was chosen with taking the research design, the amount of data being analyzed, and the complexity of the coded classification process of the study into account. In addition, the coding process is ensured to be consistent and reliable with involving 2 researchers including the author and his colleagues (LH and ADW) with a background in information technology and understanding of the Satusehat platform. This established intercoder reliability in order to achieve valid interpretation of the data [20]. After conducting our content analysis and categorizing the issues, we developed a proposed approach for each issue category as shown in Figure 4. These proposed approaches are feasible incorporated into the existing Satusehat platform architecture, potentially resulting in an interoperability design that is less burdensome for developers. By less burdensome, we mean that the design can be easily implemented by novice developers, which are commonly found in private clinics and *Puskesmas* (community health centers). This design can be referred to as the FHIR-based Satusehat platform interoperability design for private clinics and *Puskesmas*. By reducing the burden of the interoperability design, the integration of EMR systems into the Satusehat platform in Indonesia will be smoother. It is important to note that there are other factors that can influence the smooth implementation beyond the technical scope this study. For the sake of the study’s conciseness, we will focus only on the technical issues from a developer’s perspective.

### Ethical Considerations

Public Telegram group of the Satusehat developer hub contents was used for content analysis in this research. There were no any extra compensation given to conduct the analysis since the content itself is public. We acknowledge the ethical concerns that follows while analyzing data that may raise privacy issues. An original study relating to framework that can be used by researchers to consider ethical issues in web-based research also included how privacy policy and content that were analyzed on specific web-based platform should be reviewed. The collected data have to be accessible to public, focused to the content, and not the individual who posted the content [21]. In addition, taking into account the public nature of the group and also Telegram’s privacy policy relating to public chats, we believe that group members implicitly consented to having their data accessible publicly [22]. Following the logics provided in the framework and Telegram’s privacy policy, the exemption of additional ethics review is feasible for this study. This study was reviewed by the Medical and Health Research Ethics Committee Faculty of Medicine, Public Health and Nursing, Universitas Gadjah Mada-Dr. Sardjito General Hospital, Indonesia (KE/FK/0204/EC/2023).

The focus of this study is to analyze the content of the public developer hub and not on the individual who posted in the developer hub. Therefore, anonymization is applied to the data that are collected and presented in this study to ensure group participant’s data privacy and confidentiality. According to other literatures, it is stated that waiver of consent could be requested when no identifying information is presented. Also, permission of further use of the data is permitted if the data are freely available on public forum [23]. Considering aforementioned points, acquiring consent was not necessary for this research. However, it is important to get consent in general for research using data from people. In our case, it was not possible to get informed consent due to the scope of our research.

## Results

### Overview

The results of our study are divided into 2 sections for clarity and organization. The first section outlines the pain points and issues with the Satusehat platform, which were identified through content analysis of the Satusehat Social Networking Service Telegram group messages. These pain points include issues with the FHIR server, difficulties in selecting the appropriate FHIR profile, and challenges in mapping EMR data into standardized data formats such as SNOMED-CT terminology [24]. Implementing and maintaining an FHIR server may be challenging due to the interoperability, security concerns, and scalability. Selecting the appropriate FHIR profile gives difficulties in aligning with specific use cases, balancing flexibility, and considering long-term maintenance. Mapping EMR data into standardized data formats such as SNOMED-CT could be challenging due to data complexity, granularity, updates, and achieving semantic interoperability. The second section of our study presents proposed designs that aim to address the pain points identified in the first section. We believe that the proposed designs will help overcome the challenges that may arise during the implementation of the Satusehat platform in primary health care settings, such as *Puskesmas* and private clinics. These proposed designs include a federated architecture for the FHIR server to ensure reliable and distributed server load management, an FHIR writer and viewer system inspired by the successful Standardized Structured Medical Record Information eXchange (SS-MIX) system in Japan [25], and an FHIR conversion framework that integrates with our FHIR writer and FHIR viewer system.

### Pain Points

Based on content analysis of the Satusehat Social Networking Service Telegram group messages, we identified 3 main types of issues faced by developers: server issues, profile issues, and mapping issues as illustrated in Multimedia Appendix 1. Among the 3 categories, server issues had the highest population with 61 chats, followed by mapping issues with 37 identified chats and then profile issues with 9 chats. Our data collection method allowed us to focus on the issues and concerns that developers faced when integrating their EMR systems with Satusehat platform using FHIR standards.

### Proposed Design

**Figure 5.**
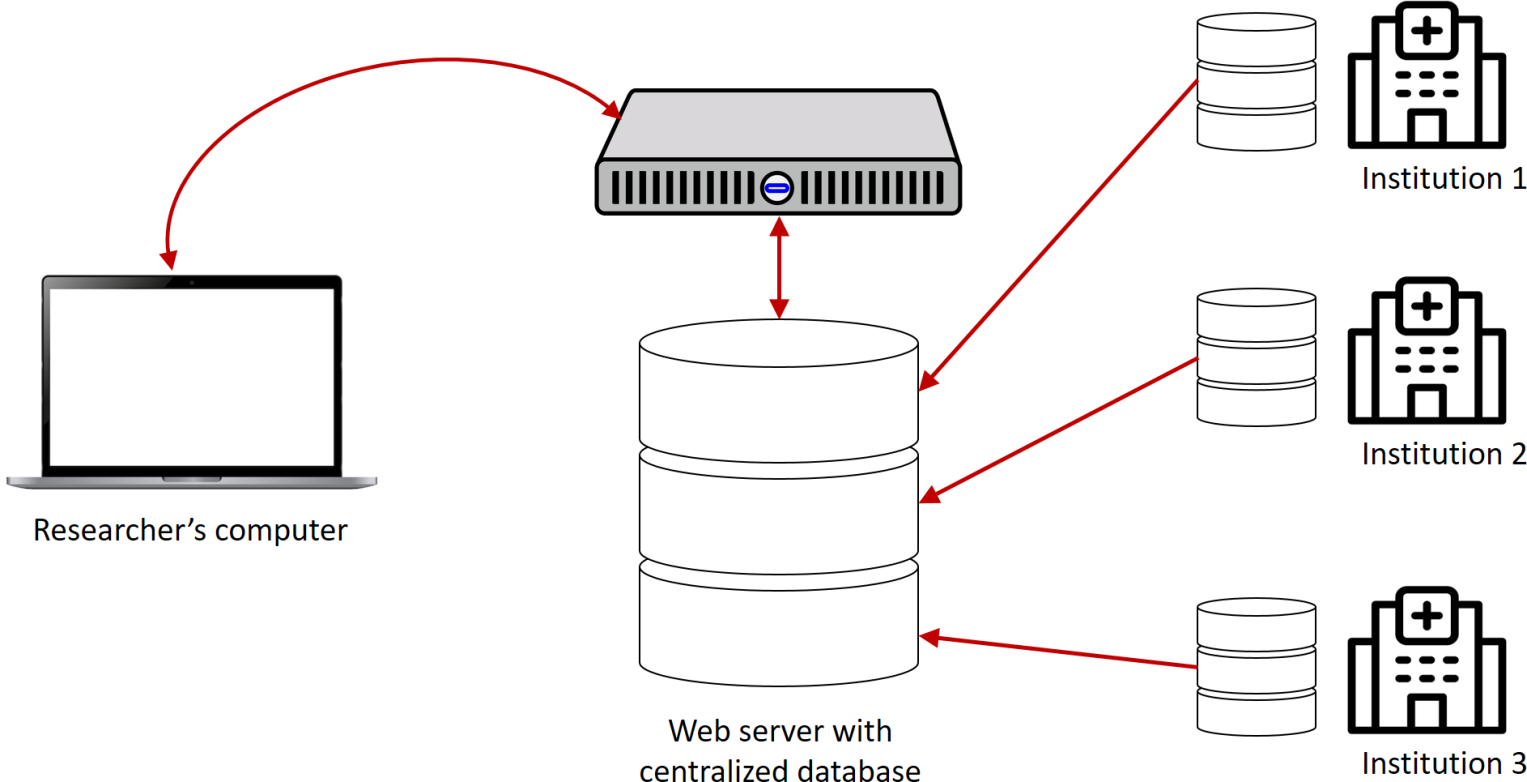
Satusehat current architecture. The platform was built on centralized architecture and database [27]. Authenticated institutions or individuals are able to access the Fast Healthcare Interoperability Resources in the central database. Writer identifies this as the root of server issue stated in developer hub.

The Satusehat platform is built on a centralized architecture, which is illustrated in Figure 5. To ensure authentication, the platform uses an OAuth server [14]. Once a user is authenticated, they can access the FHIR resources available on the platform through the FHIR API. However, based on the developers’ perspective, connecting to the Satusehat platform can be challenging. We have identified this issue as a server issue. Sometimes, the Satusehat platform server fails to respond to the user’s request, resulting in errors such as HTTP Error 401—Unauthorized, HTTP status 403—Forbidden, HTTP status 404—Not Found, and HTTP status 500—Internal Server Error [26].

**Figure 6.**
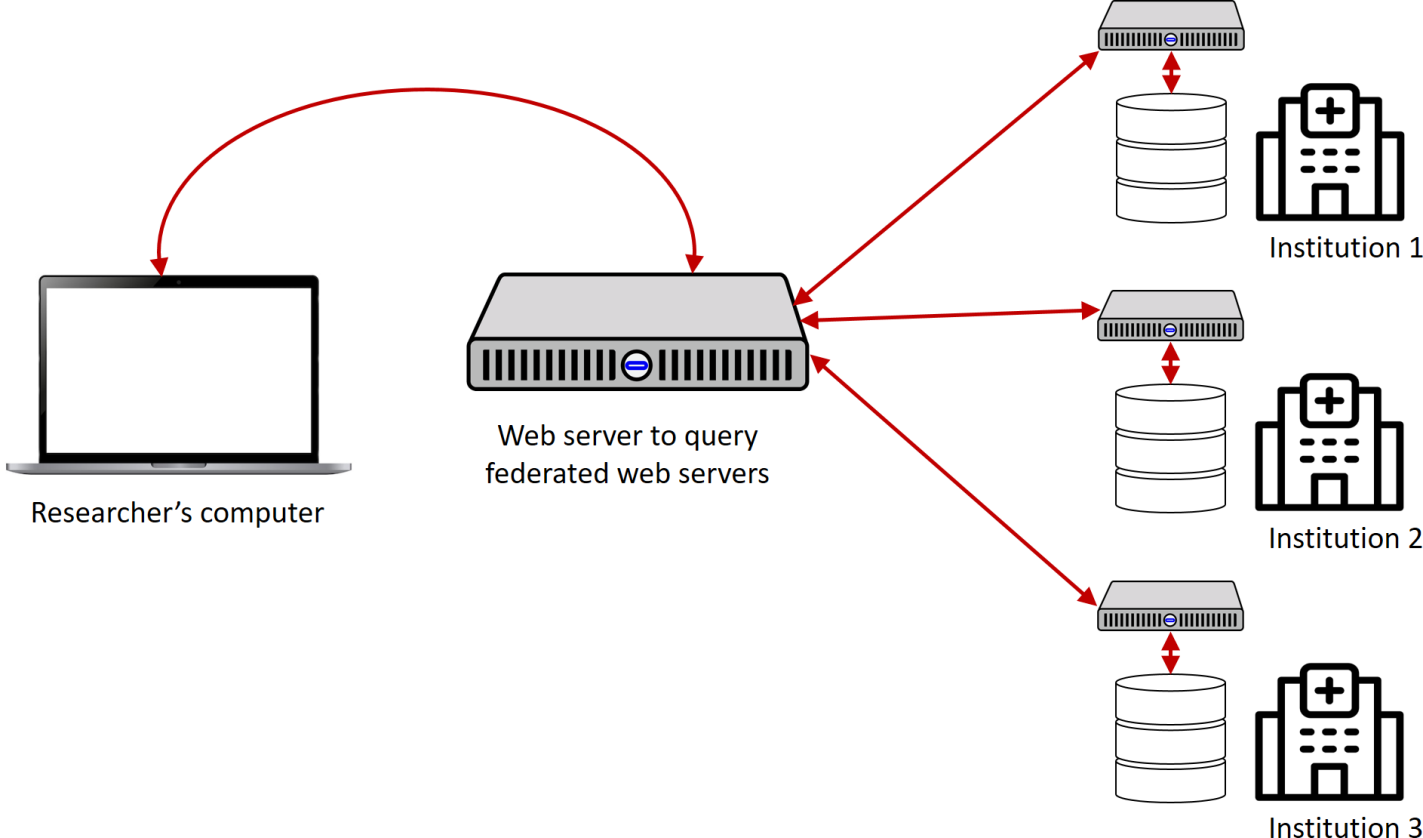
Our proposed solution: Satusehat with federated architecture [27]. The central server periodically fetch and add Fast Healthcare Interoperability Resources that are stored in local servers with the aim to enhance reliability and robustness of the platform.

To overcome this issue, we propose a federated server architecture instead of a centralized server architecture [27], as shown in Figure 6. In this approach, the FHIR resources are stored in local or regional servers that are subordinate to the central server. Periodically, the central server inquiries about the FHIR resources and copies them to the central server. This approach ensures high reliability for both the central and local or regional servers, resulting in a more robust exchange of FHIR resources. By implementing the federated server architecture, the exchange of FHIR resources will be more reliable, making it easier for authenticated users to post, get, put, and patch the FHIR resources from the Satusehat platform.

**Figure 7.**
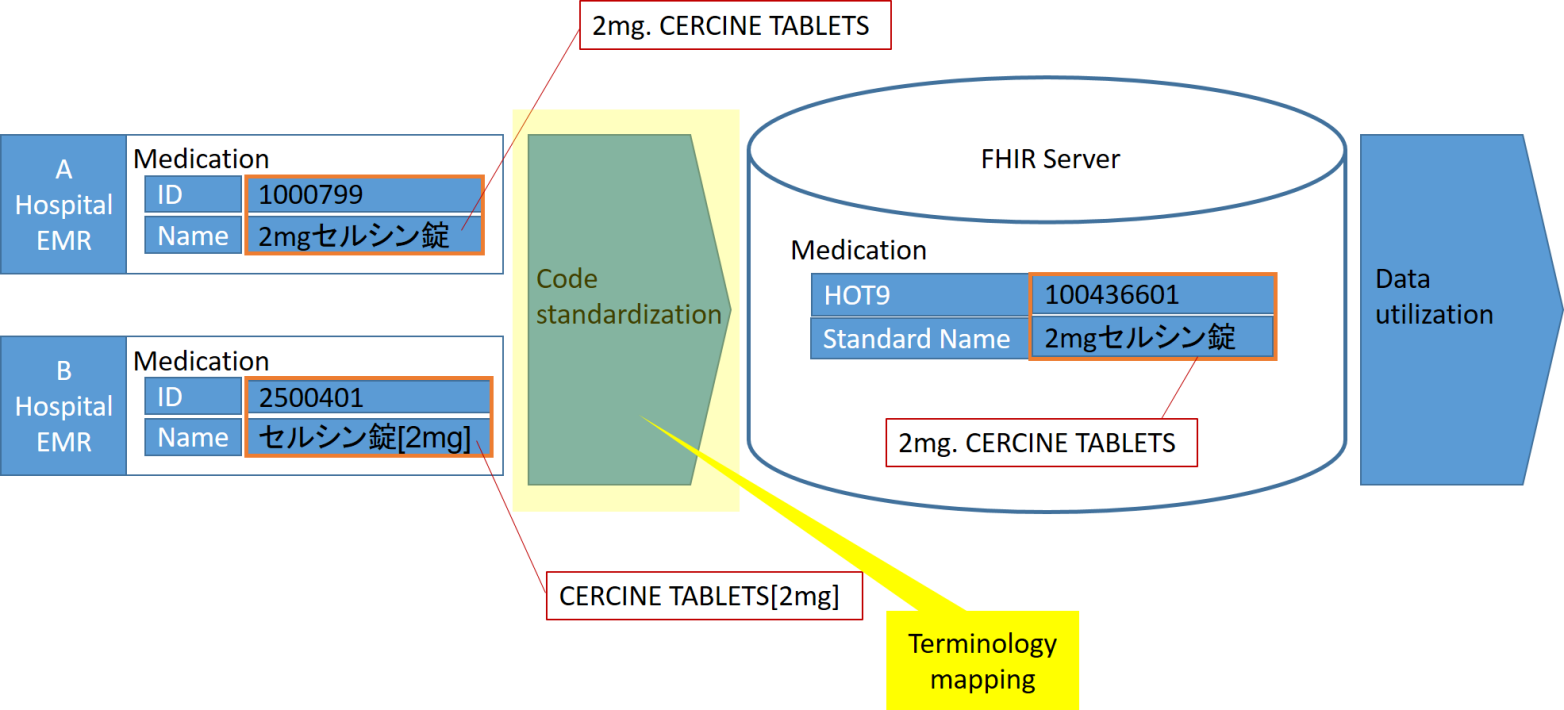
FHIR conversion framework to address mapping issues [14]. Japanese terminology for medication data, in the name attribute (2mg.CERCINE TABLETS) is preserved in hospital EMR to ensure readability. The corresponding standardized code (such as *ICD-10* [*International Statistical Classification of Diseases, Tenth Revision*]) is mapped into the FHIR server with terminology mapping. EMR: electronic medical record; FHIR: FHIR: Fast Healthcare Interoperability Resources

The issue of mapping is a significant challenge faced by developers working on the Satusehat platform. Health care facilities in Indonesia use various local terminologies, which are often in Indonesian or local tribal languages, such as Javanese. In contrast, standardized terminologies such as *ICD-10* for diagnosis and *ICD-9-CM* (*International Classification of Diseases, Ninth Revision, Clinical Modification*) for medical procedures are in non-Indonesian languages, such as English [28]. This presents a significant challenge in mapping the various local terminologies with their many variations into a single standardized terminology in English. We believe that this issue is not only unique to Indonesia but also affects other countries whose national language is not English, such as Japan, Taiwan, and Thailand [29]. To overcome this challenge, we propose an approach that is similar to the one used in Japan. The Japanese approach uses the FHIR conversion framework [30]. In this approach, the conversion is done only on the terminology code and not on the terminology text. The written terminology still uses the local language, such as Japanese, but the code follows the standardized code. This approach reduces the complexity of the mapping task significantly. The FHIR conversion framework is illustrated in Figure 7. By implementing this approach, we can ensure that the Satusehat platform can handle the mapping of various local terminologies into standardized terminologies in a more efficient and effective way.

In addition, automating the code conversion process is much simpler than the text conversion process because the variations in code are relatively small. This approach not only reduces the workload for developers but also ensures the accuracy and consistency of the mapping process. The last issue concerns the challenge of selecting and applying the correct FHIR profiles to EMR data. While there is an implementation guide available to guide users in selecting the appropriate profiles for specific use cases, such as outpatient medical resumes and immunization records, manually selecting and applying the profiles can still be difficult. To address this issue, we propose a solution that involves feeding EMR data into a system that automatically selects the correct FHIR profile and builds the content of that profile based on the input data. Similar approaches have been implemented in countries that have standardized their health data using HL7 standards, such as Japan’s use of HL7 V 2.5 to standardize various health data into a storage system called the SS-MIX [28]. The SS-MIX system has been successful in facilitating HIE in Japan. Since both SS-MIX and FHIR are from HL7, we believe that it would be feasible to adopt the SS-MIX system and modify it to use HL7 FHIR structures instead of HL7 V 2.5 structures. This is a practical solution as there are already open structures and guidelines available to implement SS-MIX. We simply need to modify the structure to use HL7 FHIR data.

## Discussion

### Principal Findings

**Figure 8.**
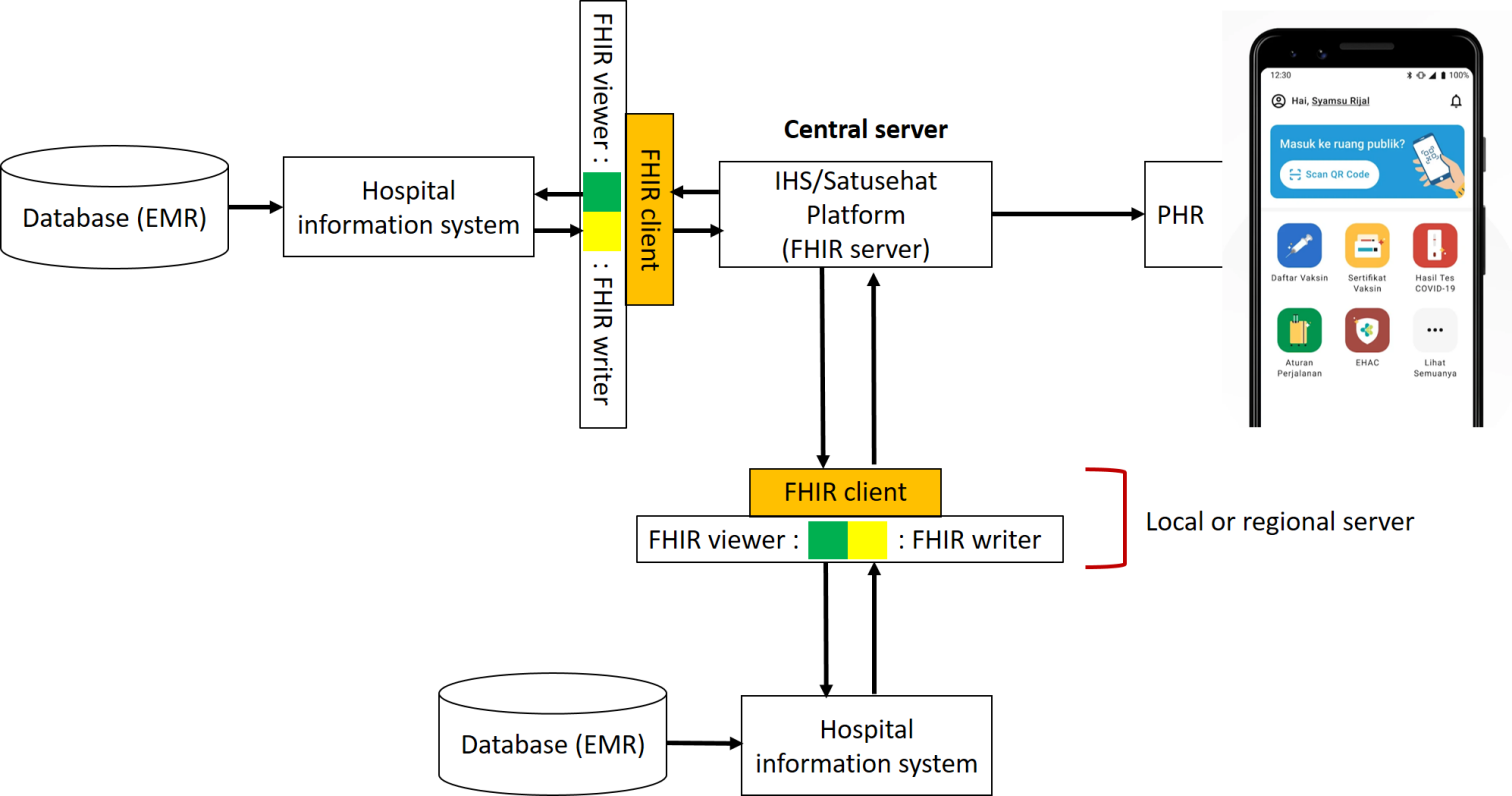
Our proposed design for FHIR-based Satusehat platform. It consists of federated architecture between central server and hospital information system in local or regional server as FHIR client, local terminology system mapping, and automated FHIR profile selection. Fast Healthcare Interoperability Resources; IHS: Indonesia Health Services; PHR: personal health record.

Server issue, mapping issue, and profile issue are identified as the most frequent problems that developers faced when integrating EMR and Satusehat with FHIR protocol. The proposed design for an interoperable FHIR-based Satusehat platform tailored to the needs of *Puskesmas* (community health centers) and private clinics is depicted in Figure 8, which illustrates the combined approaches that have led to a more reliable and user-friendly platform. The design integrates several key features, including a federated architecture that distributes the data, a data mapping system that ensures consistency across diverse health care terminologies, and an automated selection and implementation of appropriate FHIR profiles for EMR data.

To address the mapping and profile issues related to data, we propose an FHIR writer and viewer system that combines the FHIR conversion framework and the SS-MIX system. This system can be deployed on both local and regional servers to ensure accessibility for health care facilities such as *Puskesmas*, hospitals, and private clinics. To use this system, hospital information systems or EMR systems feed data into the FHIR writer subsystem. The FHIR writer then converts the local code into standardized code and selects the appropriate profile to be inserted with the standardized data. The FHIR profile, filled with standardized data, is then stored or prepared in the local or regional server and is ready to be queried by a central server. If health care facilities want to view patient data from their own facilities, their EMR system can make a request to the local or regional server to view the patient data. If the data are not available in the local or regional server, the server continues the request to the central server to retrieve the data using FHIR API. In this way, the local or regional server acts as an FHIR client, and the central server acts as an FHIR server in terms of the client-server connection [29]. Our proposed FHIR writer and viewer system provides a standardized approach to data mapping and FHIR profile selection, ensuring consistency and ease of use for health care facilities. It also allows for efficient querying of patient data across multiple facilities, increasing the interoperability and accessibility of health data. The issue of server architecture is a critical consideration in developing an efficient and reliable health information system. In particular, the ability to handle large volumes of data and ensure seamless data exchange across different health care providers is crucial. The approach to addressing this challenge lies in the use of federated architecture. Federated architecture offers a scalable and flexible solution to server issues by distributing the load across multiple servers [30]. This ensures that the server load is manageable even during peak periods, such as when there is an automatic processing of EMR data. By spreading the load, federated architecture also enhances the reliability of the system, making it more robust and resilient. Moreover, federated architecture is an ideal solution for countries such as Indonesia, which has a vast archipelago with numerous islands separated by sea. With federated architecture, health facilities in remote areas can be connected to a centralized health information system, enabling the efficient exchange of health data and improving patient care. To fully leverage the benefits of federated architecture, an automated FHIR writer or viewer system can be implemented. This system enables the automatic conversion of local codes to standardized codes, the selection of appropriate FHIR profiles, and the storage and retrieval of patient data from local and regional servers. The system can be easily deployed and customized to suit the specific needs of *Puskesmas*, private clinics, and hospitals. By using this approach, health care providers can efficiently access patient data, resulting in improved health care outcomes for patients. Although our proposed design is based on a small amount of chats in the Satusehat Telegram group, we recognize that this group is the most interactive channel for developers seeking support in integrating their EMR systems into the Satusehat platform. Therefore, despite the limited data, we believe that most of the issues related to integrating EMR systems into Satusehat using the FHIR framework have been recorded in the Satusehat Telegram group. By analyzing and addressing these issues, we were able to develop a design that effectively addresses the common challenges faced by developers. We also recognize that ongoing monitoring and analysis of the Satusehat Telegram group will be critical for identifying and addressing new issues that arise as the platform continues to evolve. Our proposed design for the FHIR-based Satusehat platform can be a valuable solution for other countries facing similar challenges in implementing HIE systems. While we drew inspiration from the design of Japan’s HIE system, which shares similar characteristics with Indonesia, such as a non-English language and multiple islands, our approach is adaptable and scalable to meet the needs of other countries. By collaborating and sharing knowledge, countries facing similar issues can learn from each other to enhance the implementation of FHIR-based platforms such as Satusehat. This approach could foster the development of a regional-level HIE system in ASEAN countries or even beyond, incorporating other Asian countries such as Japan, Taiwan, and India, which have already implemented HIE systems [31]. By working together, we are able to create a more efficient and effective HIE system that benefits all participating countries.

### Limitations

The content analysis in the study was limited into specific time frame (December 20, 2022, to January 29, 2023) and a small size sample of 107 chats. It was originated from the developer hub’s Telegram group that was managed by the DTO of Indonesia MoH. The telegram also addresses only the technical pain points from developers’ perspective to integrate their EMR to Satusehat platform. Also, the proposed approaches based on the identified pain points have not been implemented to the existing Satusehat platform.

### Future Works

After gaining an understanding of the challenges faced by the Satusehat platform and proposing an interoperability design for the platform, our next step is to develop an FHIR writer or viewer system that is tailored to the Satusehat platform. To accomplish this, we plan to collaborate with an EMR system developer for the purpose of collecting EMR data. These data will be used to train and test the FHIR writer or viewer system, to determine whether the EMR data can be standardized based on the FHIR-based Satusehat platform, and to determine whether it can be viewed back by the same EMR system. We are also interested in collaborating with researchers from Japan, whose approach to building the SS-MIX system has inspired our proposed design. By working together, we aim to create an FHIR-compliant version of SS-MIX that can not only be used in the Satusehat platform in Indonesia but also has the potential to be implemented in Japan. With this joint collaboration, the FHIR-based SS-MIX can facilitate cross-country border HIE by ensuring interoperability and seamless exchange of health data between the 2 countries.

### Conclusions

For ensuring successful implementation of the Satusehat platform, this paper proposes content analysis as a way to identify pain points that are faced by developers during development. Analyzing Satu Sehat developers’ discussions on social networking system, we have identified 3 top pain points about Satusehat platform: server issues, data mapping, and difficulties managing user profiles. We have showed how using federated server architecture, an FHIR writer and an FHIR viewer, and an FHIR conversion framework could address the mentioned issues. The proposed architecture for solving pain points gives several suggestions as a blueprint on how to reduce developer workload when committing integration, increasing the robustness of the resources exchange, and ensure accuracy and consistency on mapping and also profiling process. All of this will further help the advancement of Satusehat platform implementation in *Puskesmas* (community health centers) and private clinics in Indonesia.

## Supplementary material

10.2196/51270 Multimedia Appendix 1 Most prominent pain points perceived by developers when testing Fast Healthcare Interoperability Resources from the Telegram group (developer hub).

## Abbreviations

API: application programming interface
DTO: Digital Transformation Office
EHR: electronic health record
EMR: electronic medical record
FHIR: Fast Healthcare Interoperability Resources
HIE: health information exchange
HL7: Health Level Seven
*ICD-10*: *International Statistical Classification of Diseases, Tenth Revision*
*ICD-9-CM*: *International Classification of Diseases, Ninth Revision, Clinical Modification*
LOINC: Logical Observation Identifiers Names and Codes
MoH: Ministry of Health
PHR: personal health record
SNOMED-CT: Systematized Nomenclature of Medicine Clinical Terminology
SS-MIX: Standardized Structured Medical Record Information eXchange

## References

[1] Sonoda T (2011). Evolution of electronic medical record solutions. Fujitsu Sci Tech J.

[2] Ishigure Y (2011). Trends, standardization, and interoperability of healthcare information. NTT Tech Rev.

[3] Joyia GJ, Akram MU, Akbar CN, Maqsood MF Evolution of health level-7: a survey.

[4] Mandel JC, Kreda DA, Mandl KD, Kohane IS, Ramoni RB (2016). SMART on FHIR: a standards-based, interoperable apps platform for electronic health records. J Am Med Inform Assoc.

[5] Xiao D, Song C, Nakamura N, Nakayama M (2021). Development of an application concerning fast healthcare interoperability resources based on standardized structured medical information exchange version 2 data. Comput Methods Programs Biomed.

[6] Faulkenberry JG, Luberti A, Craig S (2022). Electronic health records, mobile health, and the challenge of improving global health. Curr Probl Pediatr Adolesc Health Care.

[7] Dullabh P, Heaney-Huls K, Hovey L (2022). The technology landscape of patient-centered clinical decision support—where are we and what is needed?. Stud Health Technol Inform.

[8] Harahap NC, Handayani PW, Hidayanto AN The challenges in integrated personal health record adoption in Indonesia: a qualitative analysis of regulatory perspectives.

[9] Ministry of Health of the Republic of Indonesia (2021). Blueprint for digital health transformation strategy 2024. https://dto.kemkes.go.id/ENG-Blueprint-for-Digital-Health-Transformation-Strategy-Indonesia%202024.pdf.

[10] Chong HY, Allotey PA, Chaiyakunapruk N (2018). Current landscape of personalized medicine adoption and implementation in Southeast Asia. BMC Med Genomics.

[11] Saputra Y, Ashila MN, Muliarini P Readiness and acceptance of electronic medical records among health professionals in indonesia.

[12] Handayani PW, Saladdin IR, Pinem AA, Azzahro F, Hidayanto AN, Ayuningtyas D (2018). Health referral system user acceptance model in Indonesia. Heliyon.

[13] Postman.

[14] Lee YL, Lee HA, Hsu CY, Kung HH, Chiu HW (2020). Implement an international interoperable PHR by FHIR—a Taiwan innovative application. Sustainability.

[15] Harbi A (2021). Health care expert’s readiness to implement National Unified Medical Records (NUMR) system in the United Arab Emirates: a qualitative study. IJCAI.

[16] Bender D, Sartipi K HL7 FHIR: an agile and restful approach to healthcare information exchange.

[17] DTO kementerian kesehatan—home page. dto.kemkes.go.id.

[18] Renz SM, Carrington JM, Badger TA (2018). Two strategies for qualitative content analysis: an intramethod approach to triangulation. Qual Health Res.

[19] Kim H, Jang SM, Kim SH, Wan A (2018). Evaluating sampling methods for content analysis of Twitter data. Soc Media + Soc.

[20] O’Connor C, Joffe H (2020). Intercoder reliability in qualitative research: debates and practical guidelines. Int J Qual Methods.

[21] Cilliers L, Viljoen K (2021). A framework of ethical issues to consider when conducting internet-based research. SA J Inform Manage.

[22] Telegram Telegram privacy policy. https://telegram.org/privacy/id?setln=en.

[23] Tripathy JP (2013). Secondary data analysis: ethical issues and challenges. Iran J Public Health.

[24] Döring M (2018). Evaluating an approach for mapping FHIR profiles to research protocols. Doctoral dissertation.

[25] Japan Association for Medical Informatics (2014). SS-MIX2 standardized storage’ explanation of the structure and guidelines for implementation ver. 1.2. https://www.jami.jp/jamistd/docs/SSMIX2/descript-implemglonSS-MIX2_V1.2.pdf.

[26] TIBCO TIBCO product documentation. docs.tibco.com.

[27] Vesteghem C, Brøndum RF, Sønderkær M (2020). Implementing the FAIR Data Principles in precision oncology: review of supporting initiatives. Brief Bioinform.

[28] Quan H, Li B, Saunders LD (2008). Assessing validity of ICD-9-CM and ICD-10 administrative data in recording clinical conditions in a unique dually coded database. Health Serv Res.

[29] Aljunid SM, Srithamrongsawat S, Chen W (2012). Health-care data collecting, sharing, and using in Thailand, China mainland, South Korea, Taiwan, Japan, and Malaysia. Value Health.

[30] (2022). Hisashi Osanai—effort to internationalize a FHIR implementation guide in Japan | devdays. www.youtube.com.

[31] Dornan L, Pinyopornpanish K, Jiraporncharoen W, Hashmi A, Dejkriengkraikul N, Angkurawaranon C (2019). Utilisation of electronic health records for public health in Asia: a review of success factors and potential challenges. Biomed Res Int.

