# Supplementary figures and images for: Fast Healthcare Interoperability Resources (FHIR)–Based Interoperability Design in Indonesia: Content Analysis of Developer Hub’s Social Networking Service
Source: JMIR Form Res. 2025 Apr 21;9:e51270. doi: 10.2196/51270 (PMC12036547; doi:10.2196/51270)

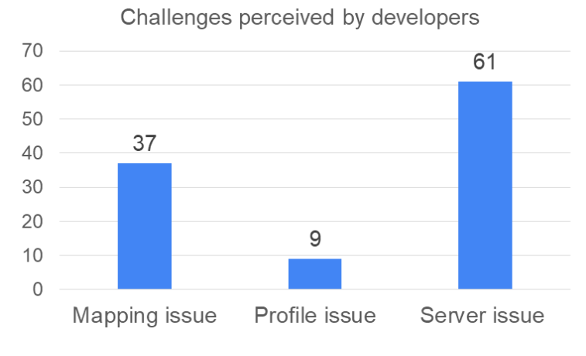

Supplement: Multimedia Appendix 1 [file formative-v9-e51270-s001.png]
